# Walking It Off: More Stressors and Perceived Stressor Control Predict More Physical Activity in Daily Life

**DOI:** 10.1093/geroni/igab046.164

**Published:** 2021-12-17

**Authors:** Erica O'Brien, David Almeida

**Affiliations:** Pennsylvania State University, University Park, Pennsylvania, United States

## Abstract

Research shows that, while the experience of stress relates to lower levels of physical activity (PA), people who perceive a greater sense of control engage in higher levels of PA. This study explores whether a sense of control specifically over stressful situations moderates the negative association between stressor exposure and PA in daily life. We used 8-day diary data from up to 1,236 participants (Age: Range = 43-91, M = 62.47, SD = 10.20) in the National Study of Daily Experiences. Somewhat contrary to hypotheses, people reported spending more time on light PA (but not moderate-to-vigorous PA) on days when they also experienced more stressors than usual. Perceived stressor control appears to magnify this effect, with people reporting even more light PA on days when they feel greater control. Initial findings suggest that a physically active lifestyle may help middle-aged and older adults cope with daily stressors.

